# Supplementary material for: Automated Machine Learning and Explainable AI (AutoML-XAI) for Metabolomics: Improving Cancer Diagnostics
Source: J Am Soc Mass Spectrom. 2024 May 1;35(6):1089–100. doi: 10.1021/jasms.3c00403 (PMC11157651; doi:10.1021/jasms.3c00403)
Supplement: Supplementary file 1 — js3c00403_si_001.pdf [file js3c00403_si_001.pdf]

Supplementary Information

Automated machine learning and explainable AI (AutoML-XAI) for metabolomics: improving cancer diagnostics.

Olatomiwa O. Bifarin[1], and Facundo M. Fernández[1,2*]

[1]School of Chemistry and Biochemistry, Georgia Institute of Technology, Atlanta, GA 30332, USA

[2]Petit Institute of Bioengineering and Bioscience, Georgia Institute of Technology, Atlanta, GA 30332, USA

*Correspondence: facundo.fernandez@chemistry.gatech.edu

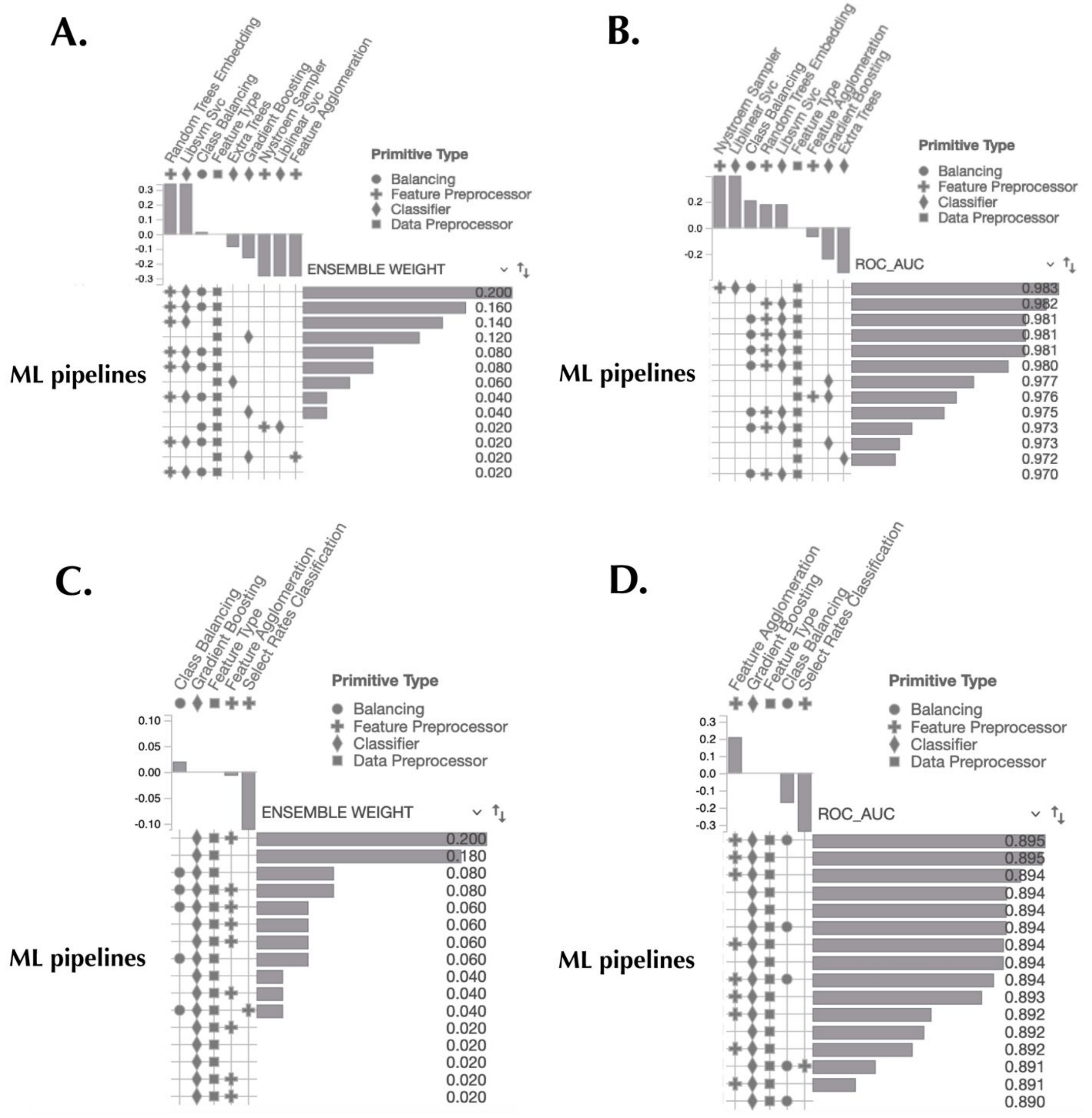

Figure S1. Pipeline profile for the ensemble model. (A) ML pipelines showing the ensemble weight contributions, RCC dataset. (B) ML pipelines showing the ROC AUC score ranking for each pipeline, RCC dataset. (C) ML pipelines showing the ensemble weight contributions, OC dataset. (D) ML pipelines showing the ROC AUC score ranking for each pipeline, OC dataset.

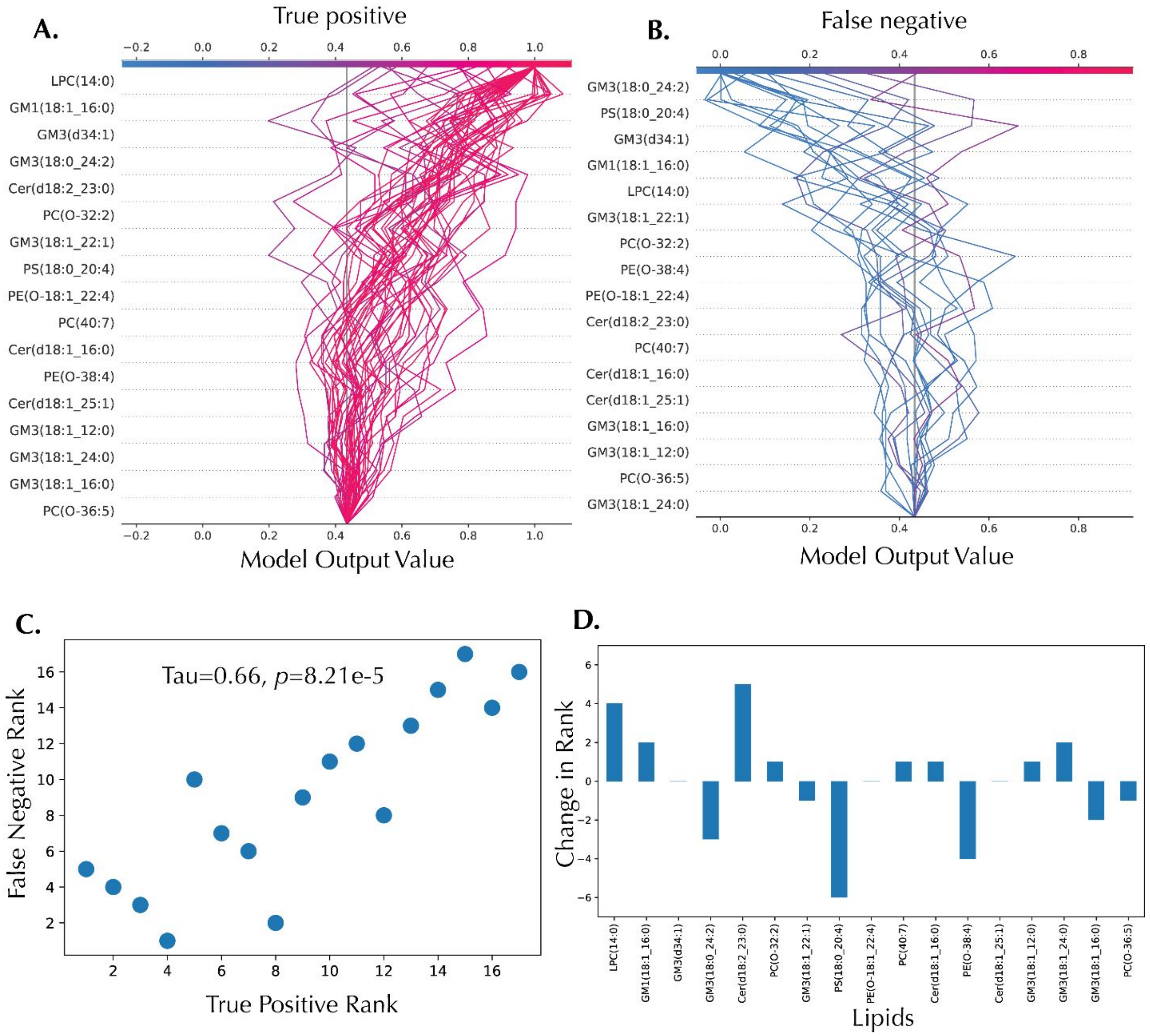

Figure S2. Error analysis decision plots for the OC Auto-sklearn diagnostic model, true positives *vs.* false negatives. (A) Decision plot for all true positive samples. (B) Decision plot for all false negative samples. (C) Feature importance rank correlation (D) Changes in feature importance rank.

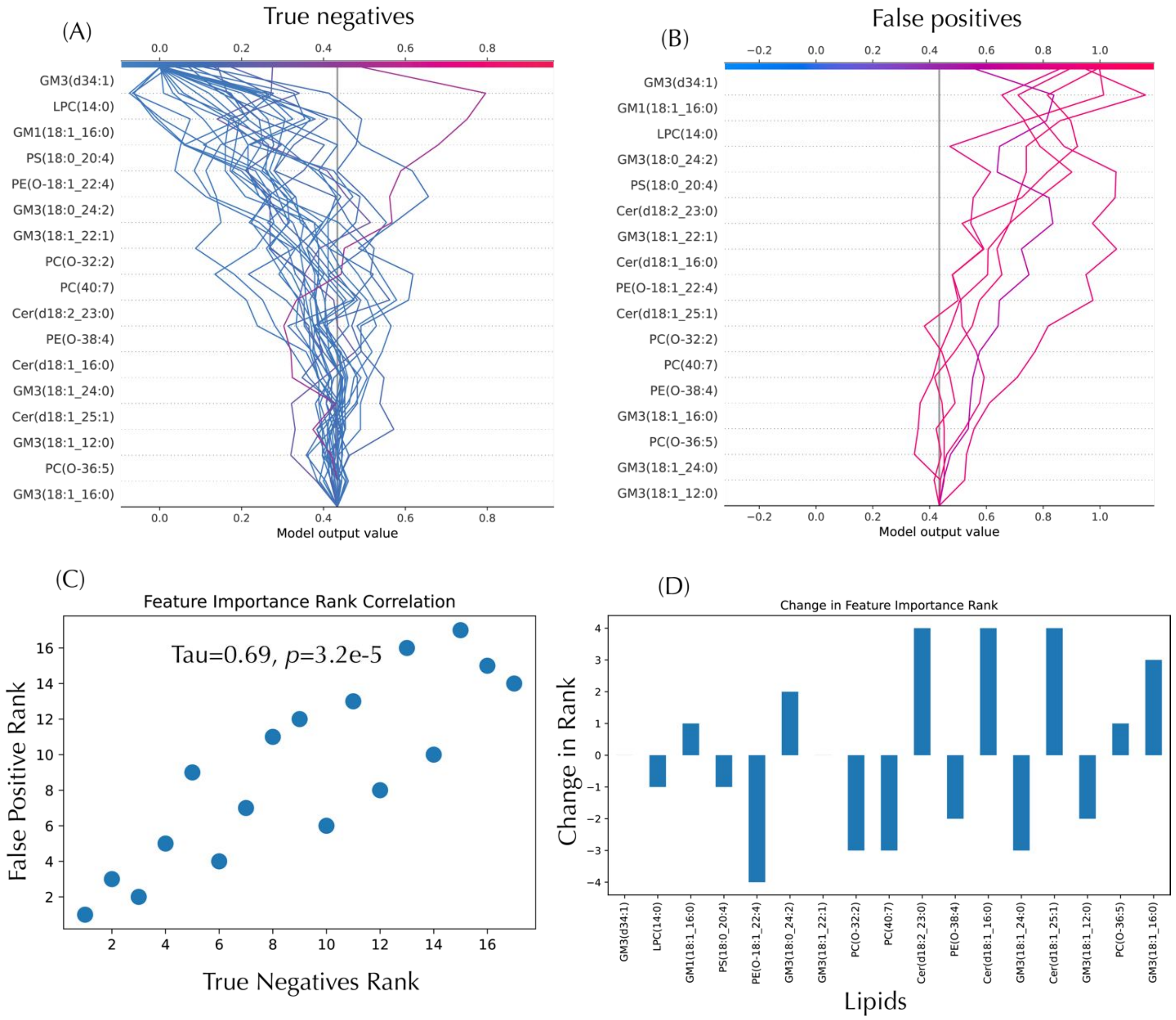

Figure S3. Error analysis decision plots for the OC Auto-sklearn diagnostic model, true negatives *vs.* false positives. (A) Decision plot for all true negative samples. (B) Decision plot for all false positive samples. (C) Feature importance rank correlation (D) Changes in feature importance rank.

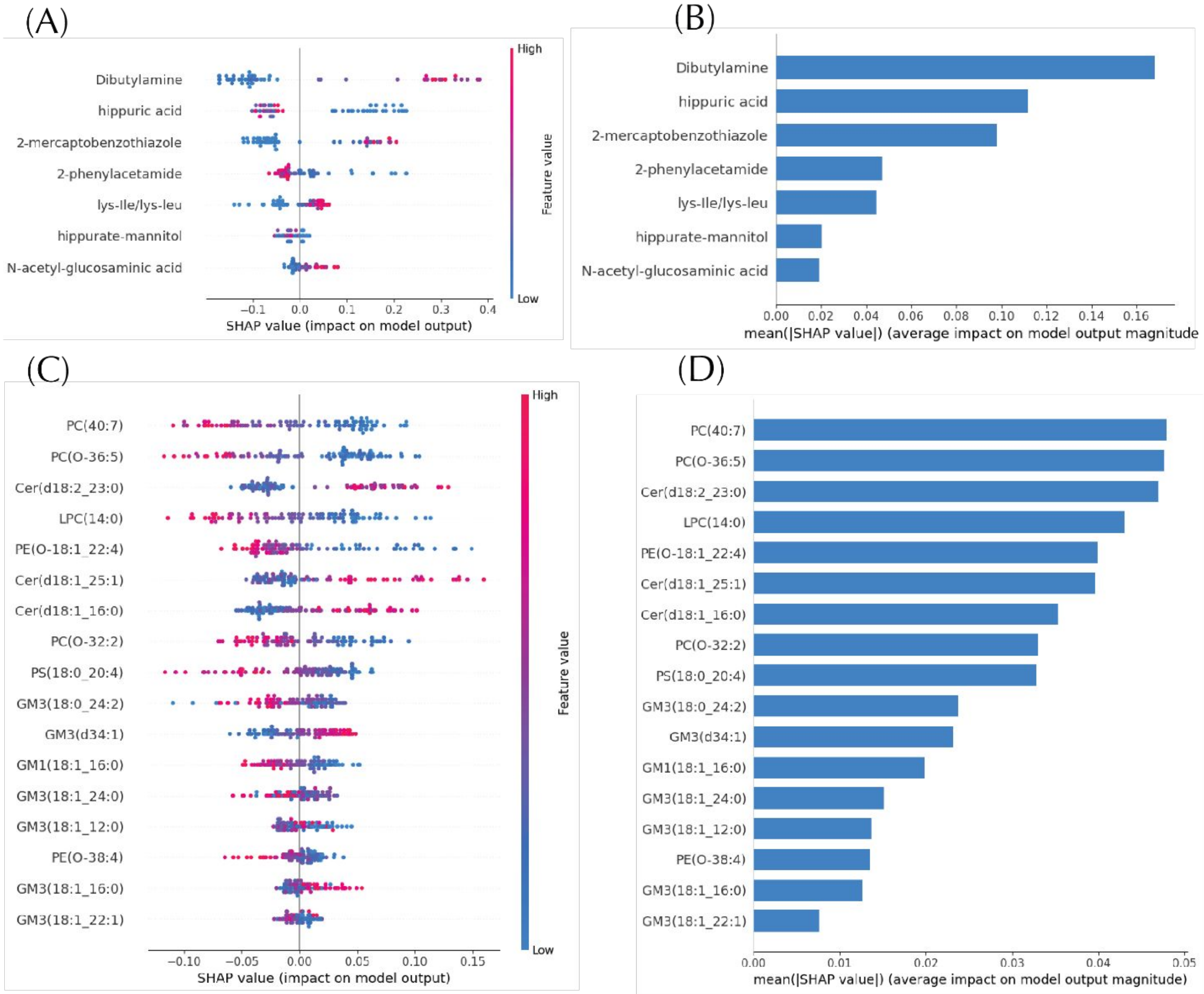

Figure S4. Machine learning interpretations of the Random Forest Model using Tree-based SHAP. (A) Beeswarm plot and (B) Summary plot for the RCC dataset. (C) Beeswarm plot and (D) for the OC dataset.

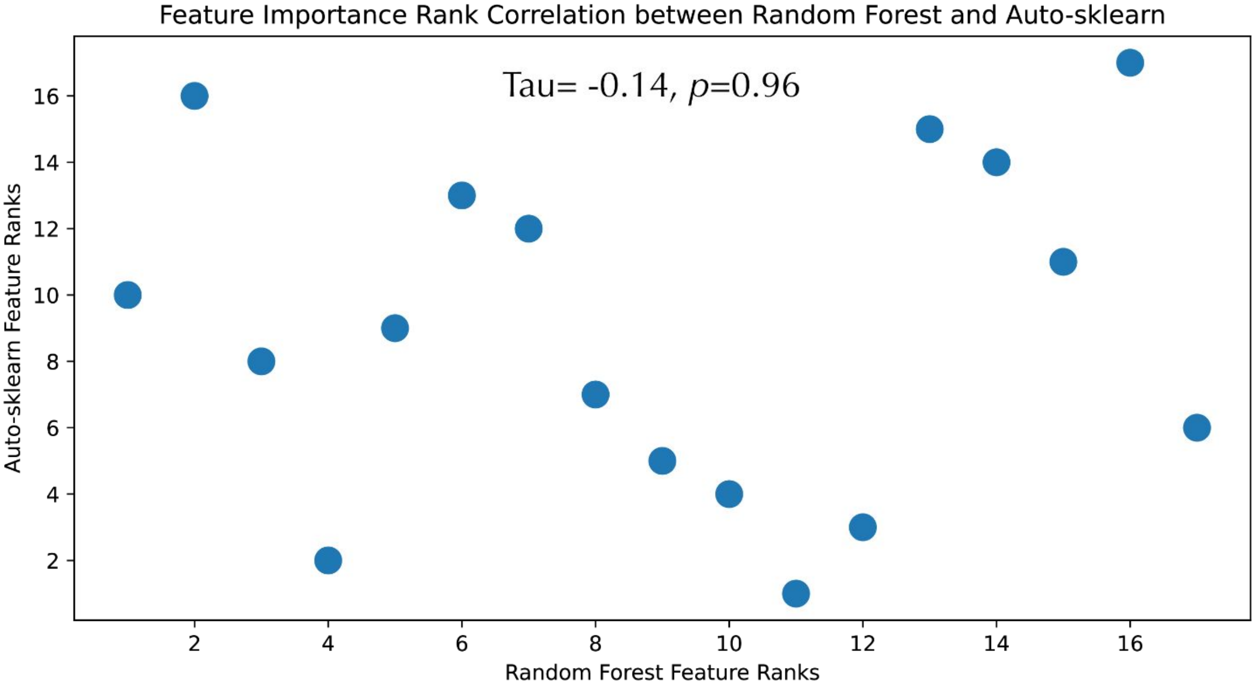

Figure S5. Feature importance rank correlation between Random Forest and Auto-sklearn for the OC dataset. *Tau* is Kendall's Tau correlation coefficient.

Table S1. Confusion matrix for RCC and OC test datasets. Presented in the Format $\begin{matrix} TN & FP \\ FN & TP \end{matrix}$ where TN = True Negative, FP = False Positive, FN = False Negative, TP = True Positive. "Negative" samples in the RCC dataset refer to healthy controls, and "Positive" samples to RCC cases. For the OC dataset, "Negative" samples denote non-OC cases, and "Positive" samples represent OC cases.

Datasets	Random Forest	SVM	*k*-NN	Autosklearn
RCC	$\begin{matrix} 32 & 2 \\ 3 & 15 \end{matrix}$	$\begin{matrix} 32 & 2 \\ 3 & 15 \end{matrix}$	$\begin{matrix} 32 & 3 \\ 4 & 15 \end{matrix}$	$\begin{matrix} 31 & 3 \\ 2 & 16 \end{matrix}$
OC	$\begin{matrix} 25 & 9 \\ 16 & 48 \end{matrix}$	$\begin{matrix} 26 & 8 \\ 18 & 46 \end{matrix}$	$\begin{matrix} 26 & 8 \\ 28 & 36 \end{matrix}$	$\begin{matrix} 28 & 6 \\ 16 & 48 \end{matrix}$
